# Smaller plants in warmer water could have implications for future Kelp forests

**DOI:** 10.1038/s41598-025-13950-z

**Published:** 2025-08-05

**Authors:** Thomas Wernberg, Karen Filbee-Dexter, Thibaut de Bettignies, Jean-Charles Leclerc, Dominique Davoult, Laurent Lévêque, Hartvig C. Christie, David C. Dyer, Robert J. Anderson, Mark D. Rothman, John J. Bolton, Kjell Magnus Norderhaug, Albertus J. Smit

**Affiliations:** 1 Institute of Marine Research, Nye Flødevigveien 20, His, 4817 Norway; 2 School of Biological Sciences, UWA Oceans Institute, University of Western Australia, Crawley, Perth, WA 6009 Australia; 3 Department of Science and Environment (DSE), Roskilde University, Roskilde, Denmark; 4 UMS Patrimoine Naturel (PATRINAT), AFB-CNRS-MNHN, 36 rue Geoffroy Saint-Hilaire, Paris, CP41, 75005 France; 5 Sorbonne Université, CNRS, UMR, Station Biologique de Roscoff, Place Georges Teissier, Roscoff, 7144 AD2M, 29680 France; 6 Departamento de Ecología, Facultad de Ciencias, Centro de Investigación en Biodiversidad y Ambientes Sustentables (CIBAS), Universidad Católica de la Santísima Concepción, Casilla 297, Concepción, Chile; 7 Marine Biology Section, Norwegian Institute for Water Research, Oslo, Norway; 8 Biological Sciences Department, University of Cape Town, Private Bag X3, Rondebosch, 7701 South Africa; 9 Department of Agriculture, Forestry and Fisheries, Private Bag X2, Rogge Bay, 8012 Cape Town, South Africa; 10 Department of Environment, Forestry and Fisheries, Private Bag X2, Vlaeberg, 8012 South Africa; 11 Department of Biodiversity and Conservation Biology, University of the Western Cape, Cape Town, South Africa; 12 South African Environmental Observation Network, Elwandle Coastal Node, Gqeberha, South Africa

**Keywords:** Climate change and ocean warming, *Laminaria*, Habitat cascades, Body size, Morphology, Bergman’s rule, Marine biology, Climate-change ecology

## Abstract

Global warming is driving contraction of species’ ranges through migration and mortality at their warm edge. However, for most species more subtle, sub-lethal changes in performance will be a more ubiquitous response to the Anthropocene. It has been suggested that reduction in body size will be a universal response to warming for cold-water species. Here we tested this hypothesis for two dominant kelp species in the northern and southern hemispheres, respectively. We tested if populations from cool and warm environments would be morphologically distinct, with warm-water populations displaying structural features indicative of sub-optimal conditions (smaller sizes). We found empirical evidence consistent with size reduction of kelp stipes, blades, and biomass of associated epiphytes from cool to warm water in both hemispheres. These changes are ecologically significant because they affect how kelps engineer their local environment, the three-dimensional habitat they create, and the associated communities they support. Reduced size of cold-water habitat forming species such as kelps may be a sublethal effect of warming that could have widespread but previously overlooked effects on the structure of ecosystems and the services that they provide.

**Supplementary Information:**

The online version contains supplementary material available at 10.1038/s41598-025-13950-z.

## Introduction

Warming has been a pervasive change to the global environment over recent decades^1^. Organisms on land and in the sea have responded with substantial changes in their distribution as conditions for persistence have changed at their range margins, either allowing expansion or driving contraction^2–4^. Research on the ecological effects of warming has had a strong focus on range dynamics and shifts at species’ range-margins (e.g.^5–8^). However, range-margins usually represent a relatively small proportion of a species’ range and along most of a species distribution global warming is unlikely to be severe enough to limit persistence. Rather, throughout the warmer half of most species’ ranges, conditions will only shift to become less optimal, not lethal (e.g.^9,10^). Consequently, sublethal effects of warming are likely to be much more prevalent than lethal effects. While not as dramatic as lethal effects, which can lead to sweeping ecosystem reconfiguration^11–13 ^the overall effects of these more subtle, sublethal changes could be substantial because of their pervasiveness.

Environmental conditions impose strong limitations on the shape, size and function of plants and animals^14,15^. This is particularly true for attached organisms which lack mobility, and therefore have to endure the circumstances that prevail where they live. While most species can survive across a broad range of environmental conditions, performance such as growth, reproduction and metabolism generally peaks within a narrow range of optimal conditions and declines outside of these^16–18^. Within a species, large body size is often considered a sign of fitness and most species attain their biggest sizes under optimal conditions^19–21^ (but see^22,23^. In contrast, persistence at suboptimal conditions is metabolically costly and leaves less available energy for growth and development^24,25^. For cool-water species, this leads to the expectation that individuals in warmer waters, where conditions are suboptimal, will be smaller than those in cooler waters.

Kelp forests are the largest vegetated coastal ecosystem^26,27^. Laminarian kelps are generally considered cool-water species that perform best in temperate, nutrient rich conditions^28–30^. Current and future ocean warming is therefore anticipated to become increasingly stressful and have negative consequences for many kelps, including species of *Laminaria*^31–33^. Many of the ecological functions of kelps, and therefore also the ecosystem services they provide, are intimately linked to their size, architecture and structural complexity^34–36^. For example, by creating a 3-dimensional structure, kelps provide a physical habitat for myriads of species living within and around the interstitial spaces created by holdfasts, fronds and epiphytes^37–40^. Habitat availability, therefore, depends to a large extent on the size of the kelp, which create the foundation for these highly diverse and productive ecosystems, providing valuable ecosystem services to humans^41^.

The temperature mediated reduction in body size of vertebrates and invertebrates is well-documented^14^ and holds for many species (i.e., Bergmann’s Rule). In contrast, fewer studies demonstrate a similar effect in marine plants, including macroalgae. Some examples do however exist. Seagrass (*Zostera marina*) shoots are largest in the middle of their latitudinal range, suggesting body size peaks where conditions are optimal^21^. In Japan, Serisawa et al.^42^ found consistently smaller kelp sporophytes (*Ecklonia cava*) in warm regions compared to cooler regions, and in eastern Australia Mabin et al.^43^ showed that both fully grown and microscopic kelp sporophytes (*E. radiata*) were smaller in warmer water. Morphological variation due to temperature differences between sites has also been documented in sugar kelp (*Saccharina latissima*) along the coasts of New York State^44^ and in Atlantic Canada^45^. In Europe, kelp (*Laminaria hyperborea*) showed reduced size and lower rates of primary production towards the warmer limit of their range^46^. Such structural readjustments can accompany physiological adaptation to sub-optimal conditions^47^. In a warming climate, the ecological consequences of shrinking body size could be pervasive and substantial—especially when habitat-forming species such as kelps are affected—because of the implied reduction in the total standing biomass (e.g.^48)^ and volume of habitable 3-dimensional structure (e.g.^49^).

**Fig. 1:**
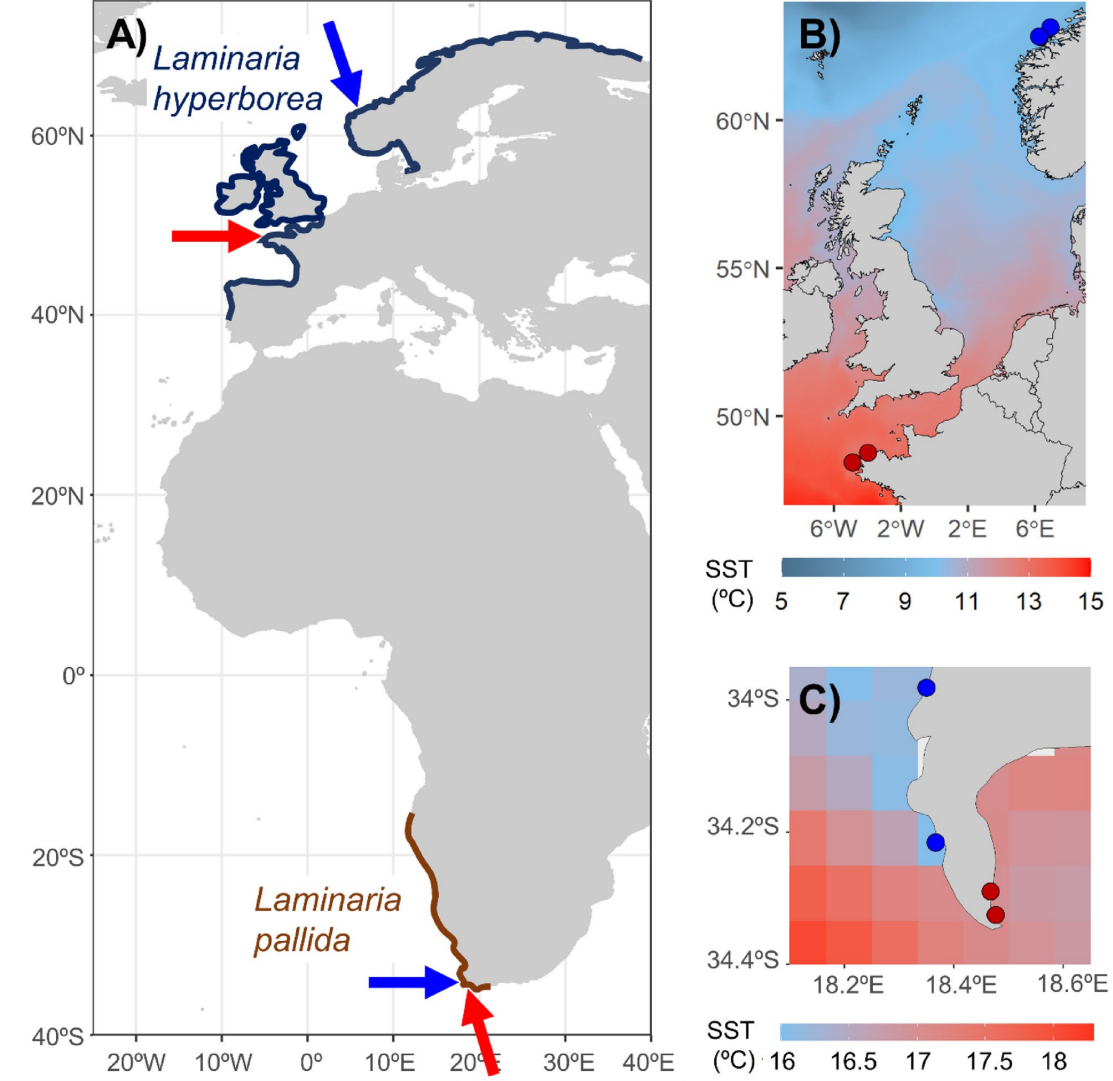
(**A**) Geographic ranges of *Laminaria hyperborea*^113^ and *L. pallida*^114^ with blue and red arrows indicating approximate location of cool and warm sites, respectively. Mean annual sea surface temperature (SST) at the study areas in (**B**) Europe and (**C**) South Africa, at cool (blue) and warm (red) sites (Bio-ORACLE from 2000 to 2014 data from Bio-ORACLE^110^. Due to upwelling and local heating and circulation close to the coast, SSTs can deviate substantially from subsurface temperature measurements (provided for each region in Table 1), which better reflect the temperatures experienced by the kelps. All comeponents of this figure were constructed by the authors using R using the mapdata package (A language and Environment for Statiscal Computing, R Core Team, R Foundation for Statiscal Computing, Vienna, Austria, 2017, https://www.R-project.org version 2.2–6, https://CRAN.R-project.org/package=mapdata) and Microsoft 365 Powerpoint version 2502.

Here we investigated morphological differences between *Laminaria hyperborea* and *L. pallida* populations growing under cool and warm conditions within their respective ranges. In the northern hemisphere, *Laminaria hyperborea* is a dominant kelp in the northeast Atlantic, occurring between Portugal and western Russia^50^. In the southern hemisphere, *L. pallida* is a common kelp species along the west coast of southern Africa^51^ (Fig. 1). These species are structurally very similar with a single rigid stipe supporting one digitated blade (Fig. S1).

**Table 1:** Summary of long-term means (and range) of sea temperature and nutrient concentrations (nitrate and phosphate) at the sampling sites (Table S1). Average C:N ratios for kelp tissue collected in spring and summer from each region*. *Light, current and nutrient data were obtained from BioOracle and averaged over 2000-2014.

**Site**	**Temperature** **(°C)**	**Light** (E m^-2^ d^-1^)	**Current velocity** (m^-1^)	**Nitrate** **(μM)**	**Phosphate** **(μM)**	**Kelp tissue C:N ratio**
*Laminaria hyperborea *Cool (Finnøy)	8.1 (3.7 – 14.9)	30.2	0.119	0.91 (0.02 – 3.37)	0.12 (0.003 – 0.33)	13.6 (11.7 – 15.0)^b^
Warm (Lanildut)	12.1 (8.7 – 16.3)^a^	31.0	0.167	0.16 (0.001 – 1.54)	0.14 (0.04 – 0.23)	19.7 (14.6 – 26.4)^c^
*Laminaria pallida *Cool (Kommetjie)	12.0 (9.3 – 15.8)	40.4	0.129	2.34 (0.78 – 5.85)	0.39 (0.23 - 0.64)	24.2 (22.3 – 26.9)
Warm (Bordjiesrif)	16.4 (12.8 - 19.8)	43.1	0.121	2.18 (0.20 – 5.76)	0.37 (0.22 - 0.62)	26.5 (22.6 – 40.8)

^a^Data from Roscoff. ^b^Average of Finnøy and Harøy. ^c^Average of Roscoff and Lanildut.

Our aim was to assess if there would be systematic differences in plant morphology between warm and cool regions, indicative of changes that could affect the structure and ecological function of kelp forests in a warmer future. Specifically, we tested the hypothesis that *L. hyperborea* and *L. pallida* from cool and warm environments would be morphologically distinct, with warm-water populations displaying structural features indicative of sub-optimal conditions. In testing this hypothesis, we identified which morphological characters contributed to differences between species and environments, and therefore also obtained an indication of the degree to which patterns of structural and ecological function might be transferable between *L. hyperborea* and *L. pallida* kelp forests.

## RESULTS

### Environmental conditions

Kelps were measured in four regions, a cool and a warm region in both the northern and southern hemisphere (Table 1, S1). In all four regions, bottom temperatures varied 4–7 °C seasonally, and, on average, the regions in South Africa were 4.1 °C warmer than the European regions (Fig. 1; Table 1) and within both hemispheres temperatures in cool regions were, on average, ~ 4 °C lower than in warm regions. Light levels were somewhat higher in South Africa compared to Norway, but within both regions the differences between cool and warm sites were minimal, as were differences in currents among regions and sites (Table 1). Nutrient concentrations (particularly nitrate) were higher in Africa compared to Europe, but were similar among cool and warm sites in each region. Conversely, C:N ratios were lower for *Laminaria hyperborea* compared to *L. pallida*, indicating higher relative nitrogen content in *L. hyperborea* tissue. C: N ratios were overlapping at cool and warm sites in both regions, and while maximum values were higher at warm sites, perhaps suggesting occasional N limitation, average values were similar at cool and warm sites (Table 1).

### Kelp morphology

**Fig. 2:**
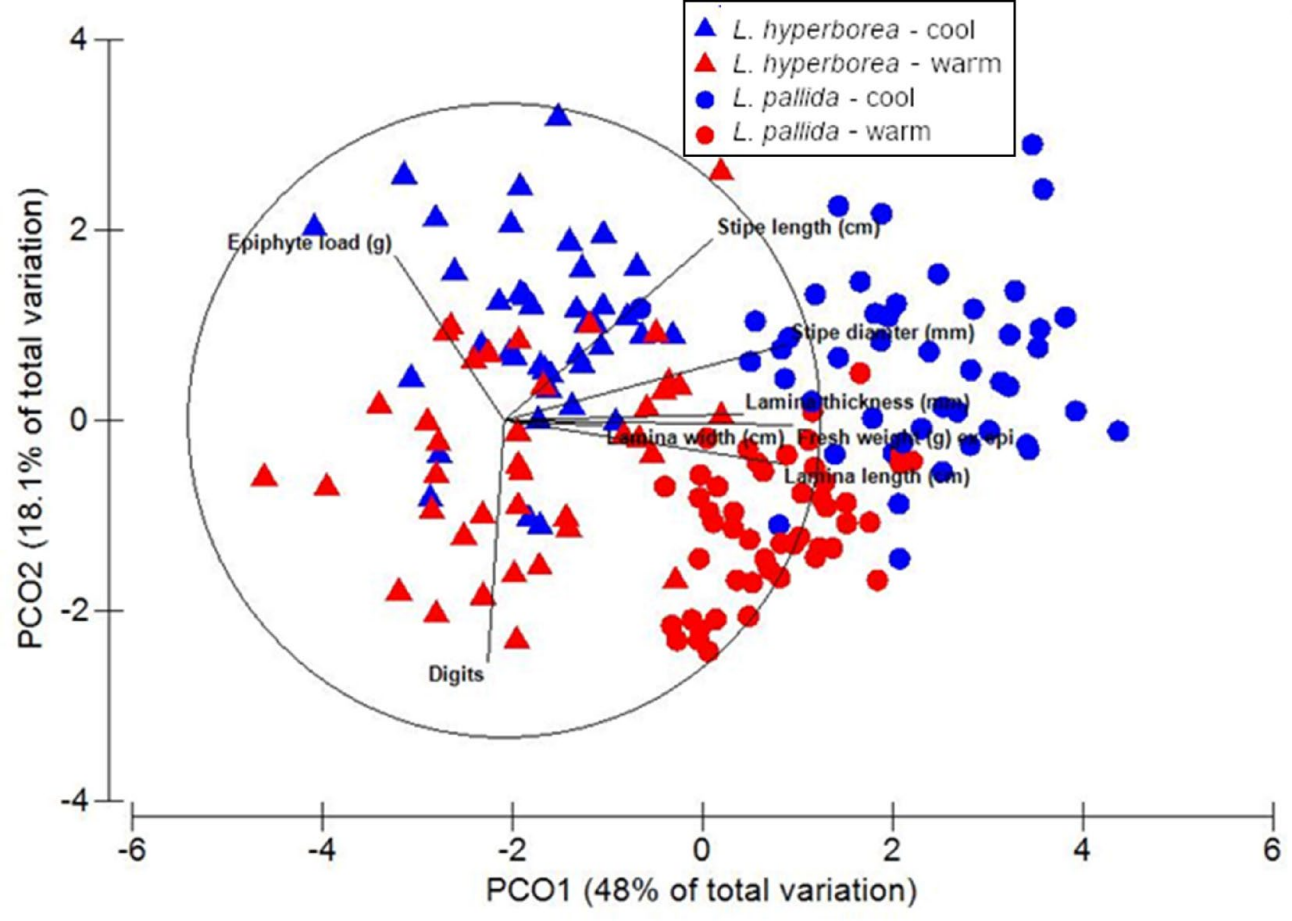
Principal Coordinates Analysis (PCO) using the Euclidian distances to visualise the group differences between morphology of *Laminaria hyperborea* (triangles) and *L. pallida* (circles) growing in cool (blue) and warm (red) regions.

**Fig. 3:**
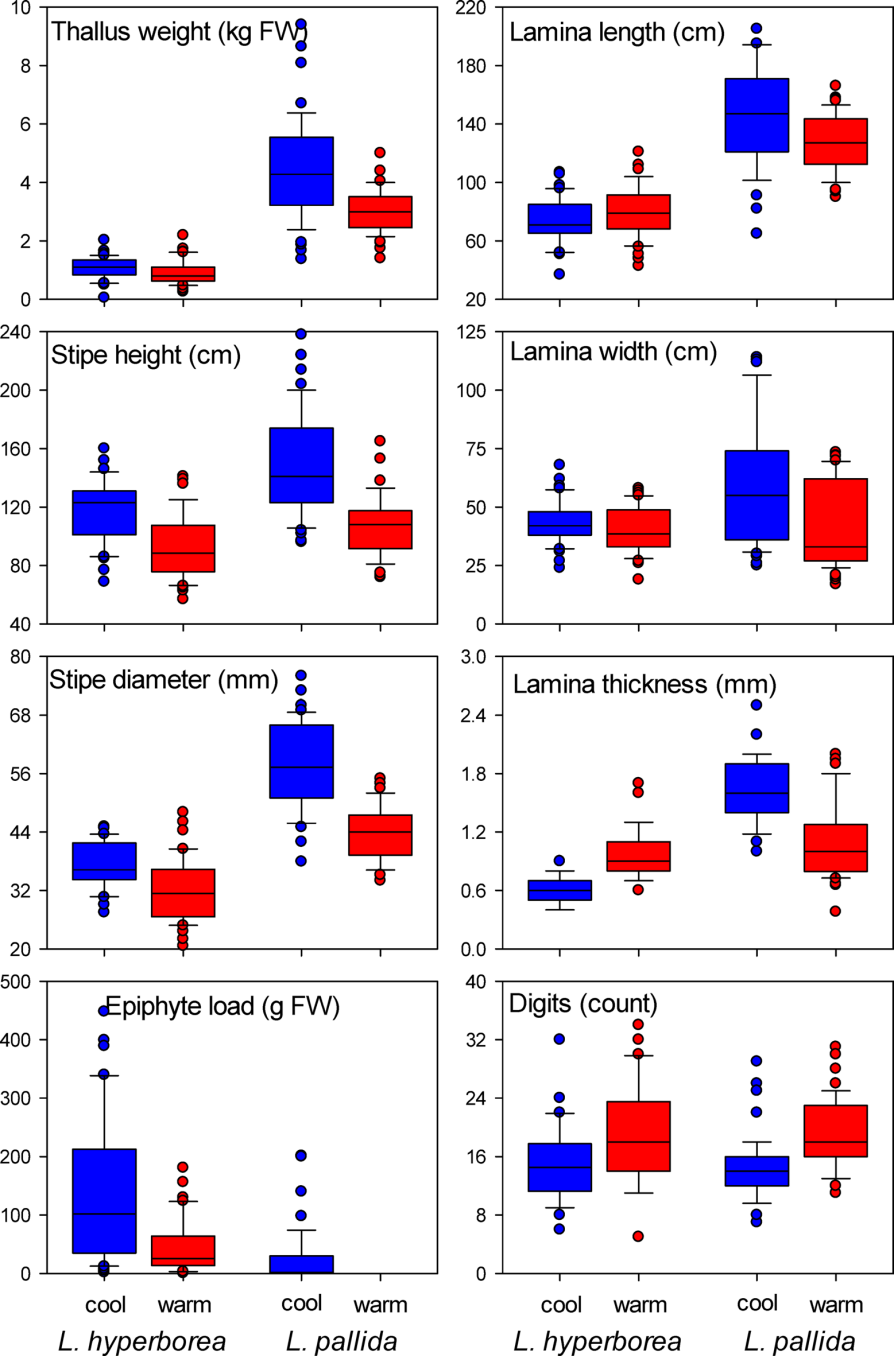
Boxplots of individual morphological characters for kelp species (*Laminaria hyperborea* vs. *L. pallida*) growing in different climates (cool vs. warm). Boxes show median, 25th and 75th quantiles, error bars show 90th and 10th quantiles, and points show outliers.

*Laminaria hyperborea* and *L. pallida* were morphologically distinct (*P* < 0.001), but both species exhibited consistent morphological differences between warm and cool regions (*P* = 0.004) over and above substantial random variation among sites (*P* < 0.001, Fig. 2, Table S2). Overall, the most pronounced differences between warm and cool sites involved the vertical structure of the kelp forest and the shape of the blades, with differences in stipe lengths, blade widths and number of digits contributing the most to morphological differences influenced by climate (Table 2). Kelps were taller and had significantly thicker stipes in cool compared to warm regions (Fig. 3). There was no difference in blade length between warm and cool regions, and thallus weight was similar for *L. hyperborea* in warm and cool regions, and significantly smaller for *L. pallida* at warm compared to cool regions (Fig. 3). Kelps had much lower total epiphyte loads in warm compared to cool regions, with no epiphytes at warm regions in Africa and 4.3 times higher total epiphyte biomass at cool compared to warm regions in Europe (Fig. 3). This was also the case for relative epiphyte load, where on average cool and warm *Laminaria hyperborea* and *L. pallida* individuals had 1.1 vs. 0.4 and 0.2 vs. 0 g epiphytes cm^−1^ stipe, respectively.

**Table 2:** Contribution of individual morphological characters to differences between species (A: *Laminaria* *hyperborea* vs. *L. pallida*) growing in different climates (B: cool vs. warm) based on SIMPER analyses. Absolute values for each character can be seen in Fig. 3.

**A**	**Morphological character**	**Species**	**B**	**Morphological character**	**Climate**
	Fresh Weight	16.3%		Stipe length	18.4%
	Lamina length	15.9%		Lamina width	18.3%
	Lamina thickness	15.5%		Digits	17.8%
	Epiphyte load	13.4%		Lamina thickness	13.0%
	Stipe diameter	13.2%		Stipe diameter	10.9%
	Lamina width	8.9%		Epiphyte load	10.0%
	Digits	8.5%		Lamina length	6.3%
	Stipe length	8.3%		Fresh weight	5.3%

## DISCUSSION

Bergmann’s rule - the tendency for individuals within a species to be smaller in warmer and larger in cooler environments, respectively - has garnered increasing interest because it predicts that a reduction in body size will be a prevalent, or even universal, response to global warming for many species^52^. Our study showed how structurally similar kelps in the northern and southern hemispheres both were smaller in warm compared to cool water areas. This pattern was evident across a suite of morphological measures, including stipe and blade dimensions, tissue thickness, biomass and epiphyte weight. These findings are consistent with both geographic patterns of smaller body sizes in warmer climates (i.e., Bergmann’s rule)^3,4,46,53^ and predicted size reductions of organisms with climate warming, such as those already seen in some populations of birds, fish, marine invertebrates, beetles and salamanders^52,54–56^.

General physiological mechanisms might explain the smaller size of kelps in warmer waters. Thermal sensitivity of growth rates and cell size has been linked to a negative relationship between development temperature and size in crustaceans^57^ and various plant species are predicted to grow slower and be smaller as temperatures rise with climate change^58,59^. As on land, where these relationships depend strongly on water availability and nutrients^60 ^in the ocean warmer water tends to hold less nutrients so kelps may grow slower.

The relationship between morphology and fitness can be complex. In general, however, kelps are more likely to reach a large body size when conditions are optimal. For example, *Laminaria hyperborea* grow the tallest stipes near the middle of their distribution range^46^. The thermal tolerance of both kelp species suggests they are in suboptimal conditions at warm sites and in more optimal conditions at cool sites. *L. hyperborea* has a wide thermal range, with optimal growth between 5 and 15 °C^61^. Ecological niche models for *L. hyperborea* shows survival in areas where temperatures are above − 0.2 °C, and do not exceed 20.9 °C in the summer or 17.7 °C in the winter^62^. According to these thermal windows, our warm sites in France, where temperatures range between 8.7 and 16.3 °C, are less optimal than our cooler sites in Norway, where temperatures range between 3.7 and 14.9 °C. For *L. pallida*, the upper and lower survival temperatures are 22–24 °C and 0 °C respectively, with optimal growth occurring between 10 and 15 °C^63^. Temperatures at our warm sites were therefore suboptimal (mean 16.4 °C; maximum 19.8 °C) compared to our cool sites (mean 12 °C; maximum 15.8 °C). In this context it is important to note that the size reduction with warming is only expected in the warm half of a species’ distribution (e.g.^52^). In the Arctic, reduced sea ice cover and warming temperatures may have the opposite effect by increasing the growth of kelps, which could lead to larger sizes and altered morphologies at cool range margins^64,65^.

Broad-scale mensurative experiments that compare ecological conditions across different extant climates, can be a powerful way to gain insight into how future climates might affect species and communities^66^. However, inferring the underlying drivers of observed differences can be challenging in comparative studies such as ours, because many potentially important factors often covary in space and time. In the northern hemisphere cool and warm sites were located at different latitudes with varying day lengths. Although variation in day length can drive strong seasonal patterns of growth^65 ^there is little evidence linking seasonal changes in day length to plant size per se. Importantly, in northern Norway annual growth of *L. hyperborea* individuals within the same age class was lower in Finnmark (~ 71^o^N) than Vega (~ 65^o^N)^67^ and an experiment on *L. hyperborea* also from Finnmark showed no differences in growth under increasing photoperiod^68^. Both of these observations are opposite to expected if longer days were driving larger individuals in cooler northern populations. In contrast, *L. hyperborea* was also found to be smaller at warmer than cooler sites across ~ 10^o^ latitude in the UK^69^. In the southern hemisphere, different nutrient regimes and currents associated with upwelling on the Atlantic coast of South Africa could have affected the growth of *L. pallida*. Nutrient concentrations and nitrogen content in kelp tissues were however similar between the warm and cool regions in the southern hemisphere, suggesting that, at the time of sampling, there were no gross differences in nutrient availability underpinning differences in plant size. Nutrients may be more important for species like *Macrocystis pyrifera* that have little to no nutrient reserves^70,71 ^compared to *Laminaria* spp., which can tolerate long periods of low nutrients^72^. Yet, short-term differences in nutrient regimes do occur in the upwelling zone, and cannot be discounted entirely. Nevertheless, the consistent response of kelp size across temperature gradients covarying with latitude (northern hemisphere) and upwelling regimes (southern hemisphere) suggest that neither day length nor nutrient conditions alone can account for the differences in kelp sizes.

Regardless of the exact mechanisms, declining size of kelps will likely have broad implications for forest and seascape structure. Kelp canopies create distinct environmental conditions by dampening water movement and reducing light levels^73–76^ which supports diverse understory communities of smaller, shade-tolerant algae and other species^34,77^. The smaller lamina sizes documented in warmer regions in this study would affect how these species ‘engineer’ the local environment such as increasing the light levels beneath the canopy and possibly altering the shaded understory communities^78^. Shorter stipes should make it easier for blades to reach and scour the sea floor during storms, which can modify community interactions in the understory^79,80^. Shorter stipes will also reduce the vertical habitat structure formed by kelps, creating less space for associated species to use for shelter and habitat^38,81^. This reduced canopy height also has implications for wave dampening effects of kelp, which may protect coasts from erosion^82^.

Kelps are highly productive species that provide food for coastal food webs through direct consumption by grazers^83–85^ or through detrital pathways^86–88^. For *L. hyperborea*, there is a strong positive relationship between total standing biomass and annual production^46,89,90^. Although it is unclear if a similar relationship holds for *Laminaria pallida*, it is highly likely that reduced standing biomass at warmer sites means that less kelp-carbon is produced and available for coastal food webs. Lower kelp biomass also means less carbon is produced, stored and available for sequestration, effectively shrinking an important potential carbon sink^46,91^.

Perhaps the most prominent pattern we observed from cool to warm sites was a large decrease in epiphyte biomass, particularly in the southern hemisphere. This could be related to the temperature tolerance of the epiphytes themselves, or be a direct consequence of the taller stipes, which provide more space for colonization. Epiphytes create important microhabitats that support high faunal abundance and diversity^92,93^. The small invertebrates associated with the epiphytes are food for fish and other species, and as a result the epiphyte biomass is tightly linked to secondary productivity^93–95^. Epiphyte abundance can also be indicative of habitat stability, suggesting kelps in cool areas live longer than kelps in warm areas^96,97^.

In conclusion, we provide evidence in support of an expected trend of shrinking organism size with warming. These temperature driven contractions in morphology of habitat forming species could be important sublethal impacts of warming with effects on ecosystem structure and the services that these habitats provide. Collapse and range contractions of kelp forests and other canopy-forming macroalgae in response to warming oceans have been predicted^31,32,98,99^ and observed on many coasts globally^11,100–104^. Yet, subtle effects of warming within the ranges of kelp are less understood^105^. These changes should be more pervasive and may be key to understanding how kelp forests respond to future climate warming. It is also unclear whether the morphological patterns we show here are the result of genetic adaptation to local climate or phenotypic plasticity, which has important implications for the rates of ecosystem change^106,107^. Species-specific responses to climate are varied and will depend on a complex interplay between multiple species and environmental changes. Although we are far from unravelling the complex interactions between environmental and ecological controls on macroalgal size, growth and communities that are required to predict general responses to warming^108 ^our results suggest that warming could lead to a contraction in kelp size at currently cool sites. We need to understand why these organisms are smaller and what these patterns will mean for biodiversity and human societies that rely on these ecosystems.

## Methods

This study compared kelps from Europe (Norway and France; *Laminaria hyperborea*) and southern Africa (Cape of Good Hope; *L. pallida*, Fig. 1). In Europe, cool and warm regions were separated along a latitudinal temperature gradient whereas in Africa, cool and warm regions were separated by the Cape Peninsula (Fig. 1, Table S1). Four accessible sites (at least 1 km apart) were sampled within each warm and cool region. This level of spatial replication was included to account for variation in site characteristics (e.g., wave exposure, water clarity, topography, herbivores). All samples were collected in spring from fully subtidal populations with a depth range across all sites of 5–15 m depth with no depth differences among temperature regimes or species (2-way ANOVA, *p* > 0.32) (Table S1).

Temperature data were obtained for each region for the period 2013–2014 where kelps were sampled. In France temperatures were recorded by an onset HOBO pendant logger at 10 m depth at a Roscoff site. In Norway temperatures were recorded by a CTD probe at 10 m on 15 separate days over the same period at the Bud hydrographic station, ca. 20 km northeast of Finnøy (http://www.imr.no/forskning/forskningsdata/stasjoner/view?station=Bud). In South Africa temperature data were recorded by Star Oddi Starmon mini temperature loggers at 8 m depth at the Bordjiesrif (warm) and Kommitjie (cool) sites, respectively. For comparison and analysis, temperatures from all regions were extracted for the same 15 dates as the measurements in Norway. Sub-surface temperatures were analysed by 2-way analysis of variance by 9,999 permutations of the residuals under a full model^109^. To visualize the temperature conditions over the range of both kelp species, mean sea surface temperatures for the larger study area were obtained from Bio-ORACLE from 2000 to 2014 (Fig. 1). Nutrient conditions at sites were compared using phosphate and nitrate concentrations obtained from Bio-ORACLE from 2000 to 2014^110,111^. We compared the long-term average, and the long-term average of the minimum and maximum concentrations of each measure per year. To further investigate these measures of available nutrients we also compared C: N ratios from kelp samples obtained from each of the four regions (Table 1). These C: N data (measured by mass spectrometry) were collected as part of other concurrent research programs in each of the four regions, and, as a result, were only available for 4 sites, one cool and one warm in each region.

At each site, 10–13 kelps were harvested by a SCUBA diver haphazardly collecting the largest individuals in sight within a 15–20 min search, targeting areas with no visible signs of kelp harvesting. Consequently, the sampled kelps represented a random subset of the largest part of the population. This sampling strategy was employed because the largest thalli are likely to reflect the maximum realised growth potential at the site and as such accounts for the many other environmental and biological factors that could act and interact to affect the mean size of the kelps (e.g., shading, tattering, herbivory and competition). Moreover, this sampling strategy also minimises potential biases from sampling insufficient numbers to describe the true mean.

Eight morphological measurements were made on all kelp thalli (Fig. S1). Total thallus wet mass (g), stipe length (cm), stipe diameter (mm), lamina length (cm), lamina width (cm), lamina thickness (mm) and number of digits. Finally, epibionts were scraped from the stipes and weighed wet (g). At these sites, the stipe epibionts were dominated by epiphytes (epiphytic seaweeds, see also^112)^.

All data analyses were done in PRIMER (version 7). Multivariate analysis of variance by permutation, PERMANOVA^109 ^was used to test for morphological differences between *Laminaria hyperborea* and *L. pallida* (Species, fixed factor) growing under cool and warm conditions (Climate, fixed factor) at four sites per combination (random factor nested in species and climate). Data were log(*x* + 1) transformed and normalised to down-weigh extreme values and to put all measures on a common scale, respectively, before 9,999 unrestricted permutations of the raw data. A similarity percentage (SIMPER) analysis was done to identify the contribution of each morphological variable to differences between Climate and Species. A Principal Coordinates Analysis (PCO) was applied using Euclidian distances in order to visualise the group differences.

## Supplementary Information

Below is the link to the electronic supplementary material.

Supplementary Material 1

## Data Availability

All data is available from the corresponding author on request.
