# Supplementary material for: Smaller plants in warmer water could have implications for future Kelp forests
Source: Sci Rep. 2025 Aug 5;15:28616. doi: 10.1038/s41598-025-13950-z (PMC12326019; doi:10.1038/s41598-025-13950-z)
Supplement: Supplementary file 1 — Supplementary Material 1 [file 41598_2025_13950_MOESM1_ESM.docx]

The following supplement accompanies the article

**Smaller plants in warmer water could have implications for future kelp forests**

Thomas Wernberg^*^, Karen Filbee-Dexter, Thibaut de Bettignies, Jean-Charles Leclerc, Dominique Davoult, Laurent Lévêque, Hartvig C. Christie, David C. Dyer, Robert J. Anderson, Mark D. Rothman, John J. Bolton, Kjell Magnus Norderhaug, Albertus J. Smit

* Correspondence: [thomas.wernberg@uwa.edu.au](mailto:thomas.wernberg@uwa.edu.au)


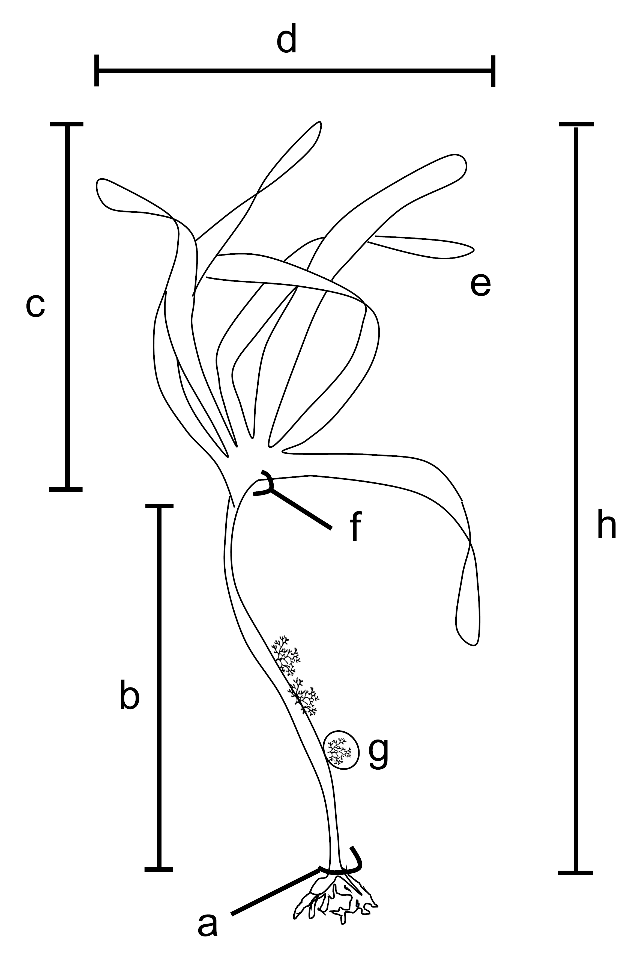


**Figure S1.** Morphological measurements taken from *Laminaria hyperborea* and *L. pallida*: stipe diameter (a), stipe length (b), lamina length (c), lamina width (d), number of digits (e), lamina thickness (f), epiphyte weight (g) and total thallus weight (h).

**Table S1.** Name and location of each study site.

| **Site** | **Temperature regime** | **Depth** | **Latitude** | **Longitude** | **Sampling period** |
| --- | --- | --- | --- | --- | --- |
| ***Laminaria pallida*** |  |  |  |  |  |
| Cape Point, South Africa | Cool | 14.1 m | 34.36192 S | 18.50003 E | October 2014 |
| Olifantsbos, South Africa | Cool | 6.0 m | 34.22800 S | 18.36959 E | October 2014 |
| Kommetjie, South Africa | Cool | 7.0 m | 34.136750 S | 18.32000 E | October 2014 |
| Sea Point, South Africa | Cool | 8.5 m | 33.91847 S | 18.38217 E | October 2014 |
| Buffelsbaai, South Africa | Warm | 8.5 m | 34.32537 S | 18.46638 E | October 2014 |
| Rooikrans, South Africa | Warm | 7.0 m | 34.34333 S | 18.47997 E | October 2014 |
| Bordjies, South Africa | Warm | 8.7 m | 34.31292 S | 18.46327 E | October 2014 |
| Roman Rock, South Africa | Warm | 5.6 m | 34.18153 S | 18.46056 E | October 2014 |
| ***Laminaria hyperborea*** |  |  |  |  |  |
| Harøy 1, Norway | Cool | 6.5 m | 62.73902 N | 6.32150 E | April 2014 |
| Harøy 2, Norway | Cool | 6.8 m | 62.74473 N | 6.34892 E | April 2014 |
| Finnøy 1, Norway | Cool | 7.5 m | 62.82373 N | 6.55176 E | April 2014 |
| Finnøy 2, Norway | Cool | 8.6 m | 62.81760 N | 6.51851 E | April 2014 |
| Lanildut 1, France | Warm | 7.6 m | 48.47328 N | 4.77997 W | April 2014 |
| Lanildut 2, France | Warm | 6.8 m | 48.49243 N | 4.79100 W | April 2014 |
| Roscoff 1, France | Warm | 12.6 m | 48.74030 N | 3.96772 W | May 2014 |
| Roscoff 2, France | Warm | 6.7 m | 48.72612 N | 3.96571 W | May 2014 |

**Table S2.** Multivariate analysis of variance (PERMANOVA) testing for morphological differences between *Laminaria* *hyperborea* and *L. pallida* (Species, fixed factor) growing under cool and warm conditions (Climate, fixed factor) at 4 sites per combination (random factor nested in Species and Climate). 10-13 individuals were collected at each site. Euclidian distances log(*x*+1) transformed and normalised before 9,999 unrestricted permutation of the raw data. Significant *p*-values (P<0.05) are highlighted in bold.

| **Source of variation** | **df** | **MS** | **Pseudo-F** | ***p*(perm)** | **Components of variation** |
| --- | --- | --- | --- | --- | --- |
| Species | 1 | 490.4 | 23.2 | **0.0001** | 31.8% |
| Climate | 1 | 140.2 | 6.6 | **0.0041** | 16.0% |
| Species x Climate | 1 | 52.0 | 2.5 | 0.0908 | 11.5% |
| Site(Species x Climate) | 12 | 21.3 | 7.6 | **0.0001** | 17.8% |
| Residual | 160 | 2.8 |  |  | 22.9% |
